# Supplementary material for: Phylointeractomics reconstructs functional evolution of protein binding
Source: Nat Commun. 2017 Feb 8;8:14334. doi: 10.1038/ncomms14334 (PMC5309834; doi:10.1038/ncomms14334)
Supplement: Supplementary Information — Supplementary Figures, Supplementary Tables and Supplementary References [file ncomms14334-s1.pdf]

Supplementary Figure 1

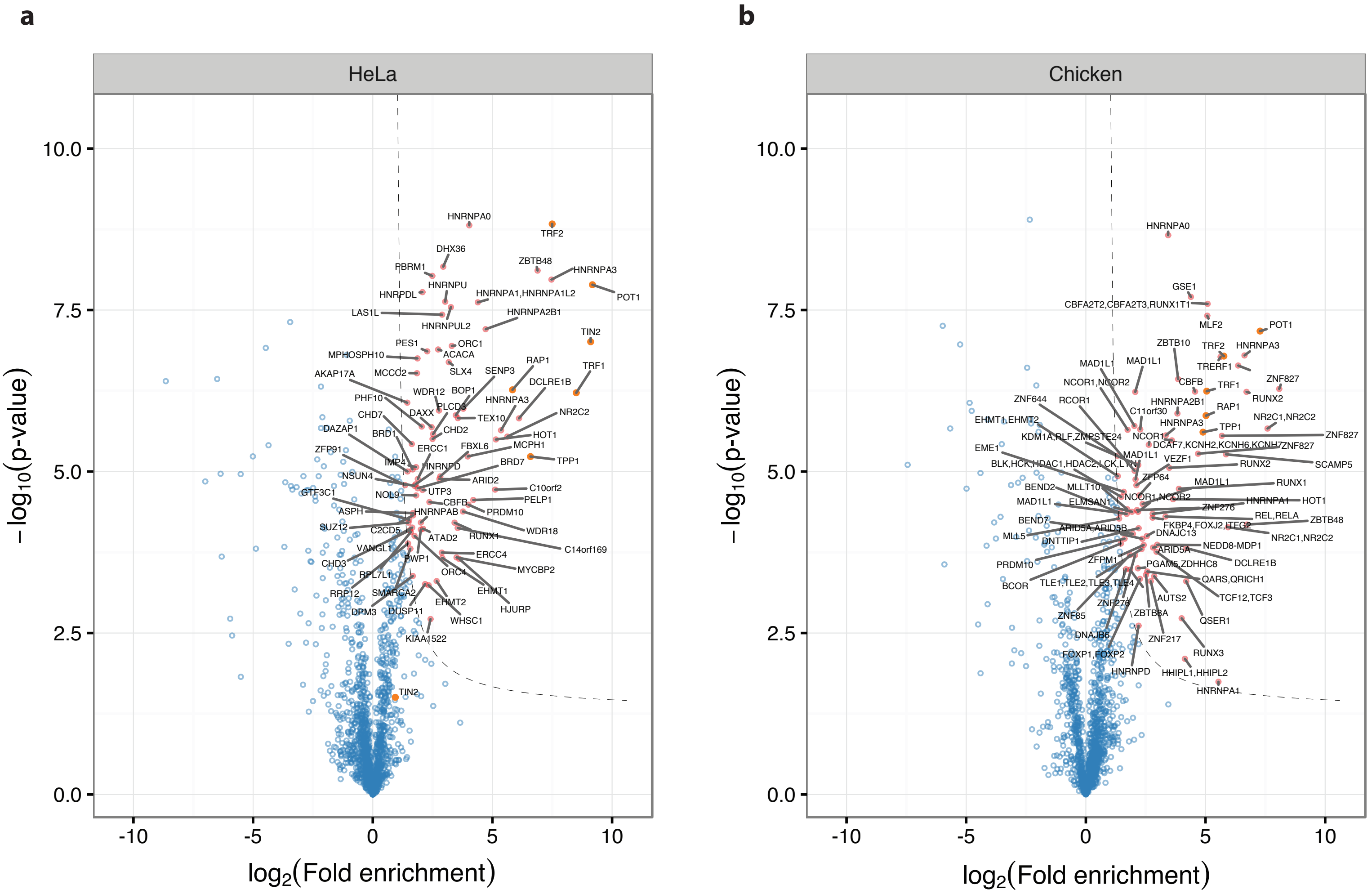

Supplementary Figure 1: Quantitative label-free DNA interaction screen with human HeLa and chicken 6C2 cells

DNA pull-downs were performed as for the evolutionary screen shown in Fig. 1. Volcano plots for (a) HeLa and (b) 6C2 cells. Specifically enriched proteins (red circles) are distinguished from background binders (blue circles) by a two-dimensional cut-off with S0=0.6 and $p<0.05$ identical to the one used in the initial screen. Detected members of the shelterin complex (TERF1, TERF2, TIN2, TPP1, RAP1 and POT1) are highlighted (filled orange dots) and all specifically enriched proteins are annotated.

Supplementary Figure 2

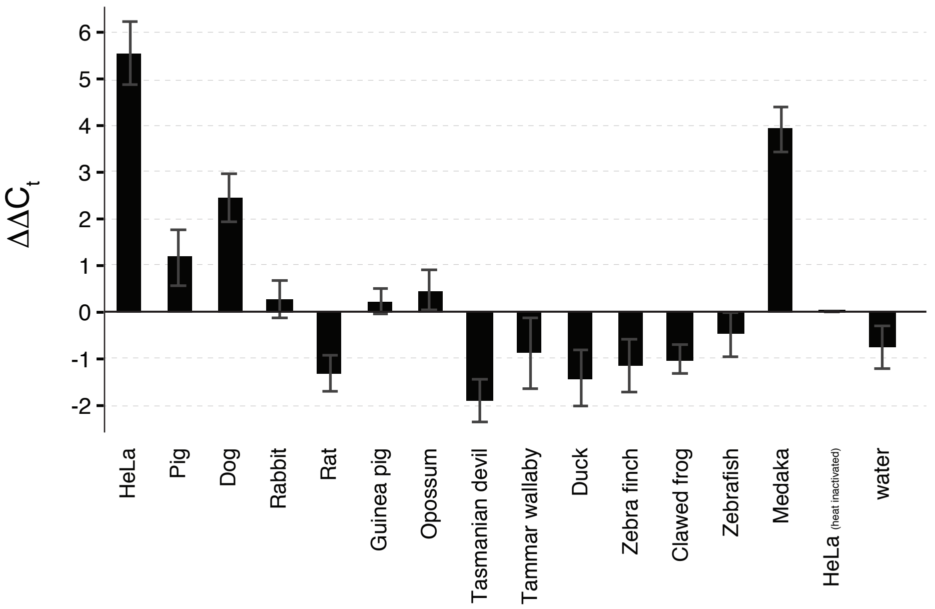

Supplementary Figure 2: Quantitative telomerase activity detection
The presence of telomerase activity in each cell line as listed in Fig. 2a was determined based on a quantitative TRAP assay. HeLa cells served as a positive control for a telomerase-positive cell line and heat-inactivated HeLa extracts were used as a minimal threshold to determine telomerase-positive cells. Differences in Ct values from the quantitative PCR measurements are displayed. Rabbit, guinea pig and opossum are considered putatively positive due to a minor deviation (<0.5ΔΔCt) whereas pig, dog and medaka show clear telomerase activity. Error bars represent standard deviations (n=4).

Supplementary Figure 3

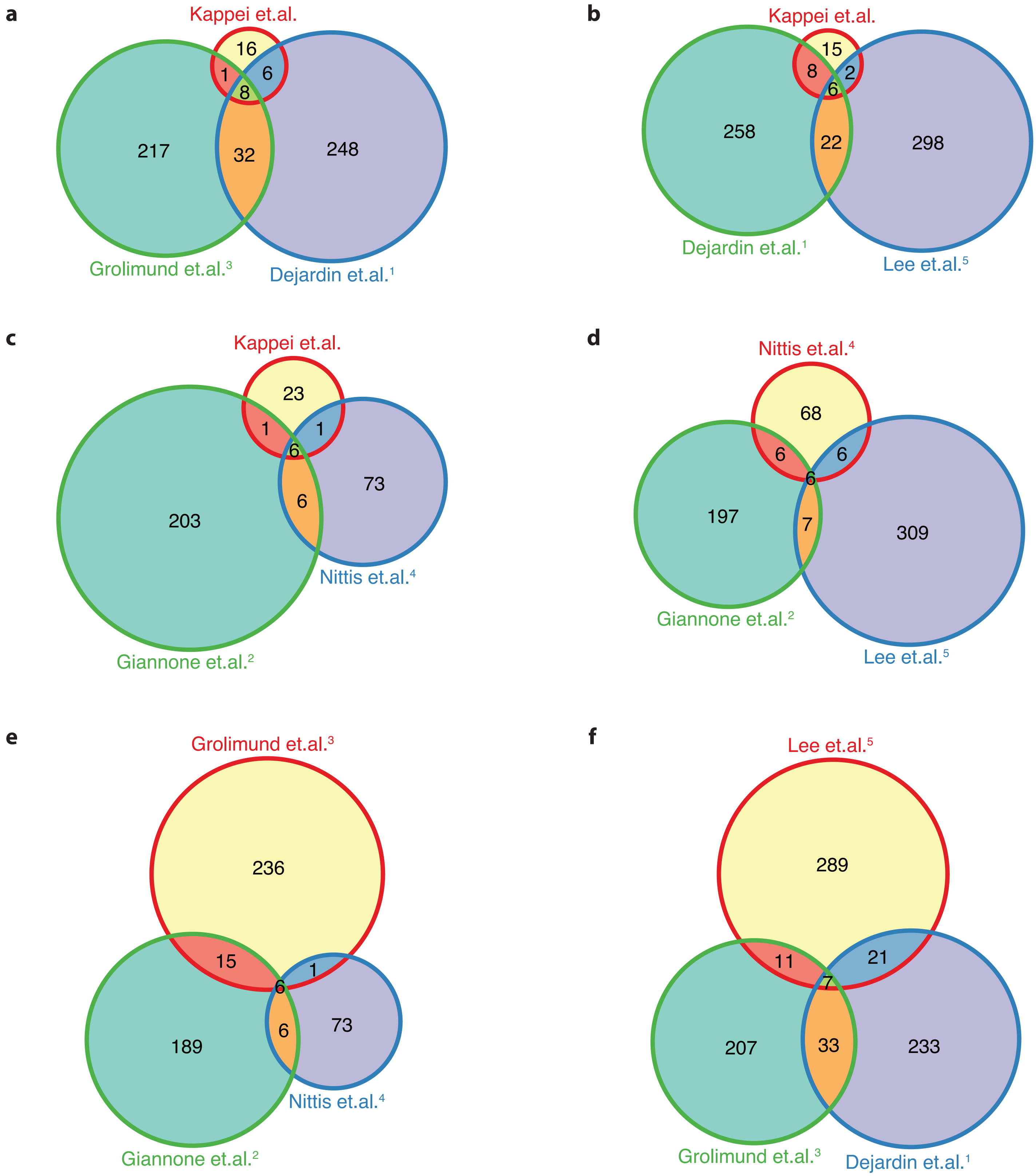

Supplementary Figure 3: Venn diagram comparison of telosome screens
The list of telomeric factors from various screens[1-5] was obtained from the TeloPIN database[6] and the overlap was calculated based on NCBI accession numbers. Numbers in the Venn diagrams (a-f) represent number of proteins that are unique or overlapping between the corresponding studies. Please note that all studies share the six shelterin proteins as a common set of factors.

Supplementary Figure 4

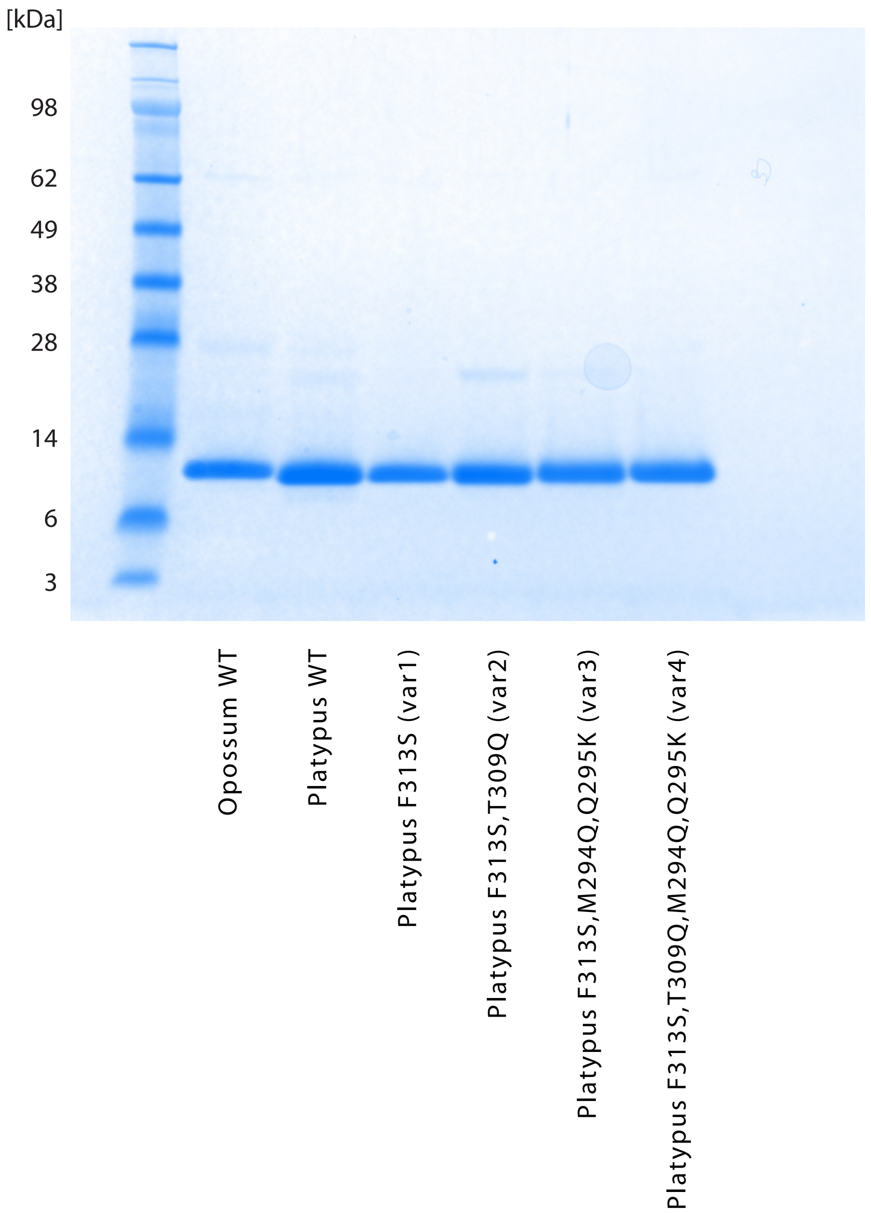

Supplementary Figure 4: Coomassie blue gel of purified TERF1 DBDs
Representative Coomassie blue gel picture of the purified TERF1 DBDs used in Fig. 3e and Fig. 3f. 5 µg of each domain were loaded on the gel. All domains show high purity and migrate at the expected molecular weight.

Supplementary Figure 5

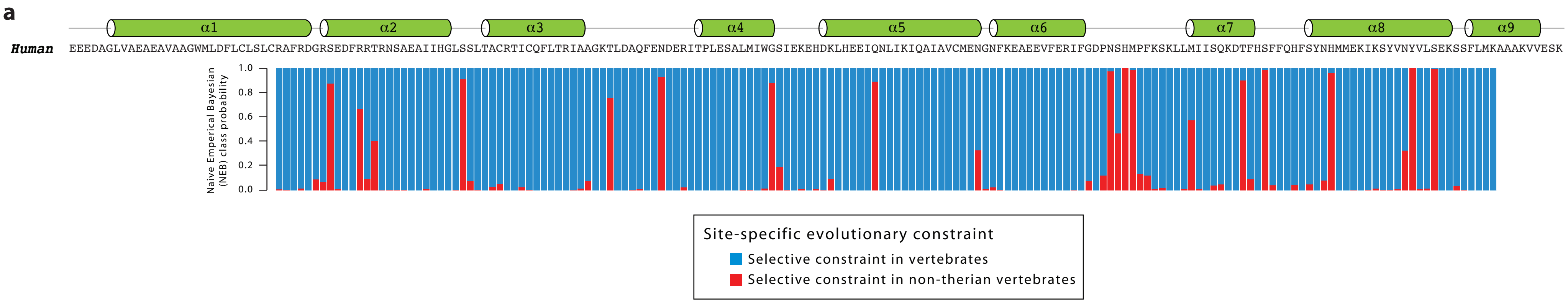

b

Model D results TERF1 TRFH domain

Site class	0	1	2
Proportion	0.38	0.51	0.11
d_N/d_S branches (blue)	0.07	0.30	ω_3=1.39
d_N/d_S branches (red)	0.07	0.30	ω_4=0.42

Model D vs M3 ($\omega_3=\omega_4$): P=0.00009 (χ^2, d.f.=1)
Model D vs D neutral (ω_3=1): P=0.06 (χ^2, d.f.=1)

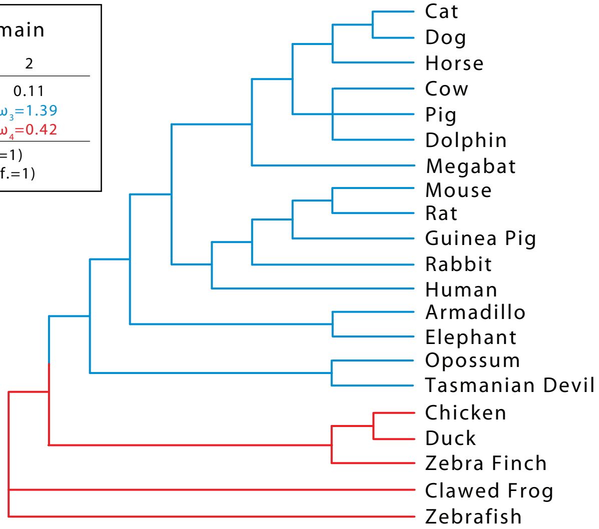

Supplementary Figure 5: PAML statistical analysis for the TERF1 TRFH domain

(a) Sequence of the human TERF1 TRFH domain. A schematic representation of the domain structure with nine α-helices (green) is shown. Below each residue is a quantitative representation of the Naive Empirical Bayesian class probability used for the branch-site modeling. Red represents selective constraints in non-therians and blue selective constraints in vertebrates. (b) Substitution rates were calculated using PAML7 to obtain the non-synonymous to synonymous substitution rate ratio (dN/dS=ω). ω values <1, =1, and >1 indicate purifying selection, neutral evolution, and diversifying (positive) selection, respectively. A branch-site model (model D) was applied and compared to a homogeneous site model (discrete Model M3) and to a Model D that assumes neutral evolution for a predefined set of branches. The phylogenetic tree represents 21 vertebrate species with available full TERF1 TRFH domain sequences that were included in this analysis.

Supplementary Table 1: PAML statistical analysis showing no significant difference in the model comparison between TERF2 homeobox domain sites. Complete TERF2 DBD domain sequences were retrieved for the following 24 vertebrate species and used for this analysis: cat, dog, horse, cow, pig, dolphin, megabat, mouse, rat, hamster, guinea pig, rabbit, human, armadillo, sloth, opossum, tammar wallaby, tasmanian devil, platypus, chicken, duck, zebra finch, clawed frog and zebrafish.

**Model D results TERF2 myb**			
Site class	0	1	2
Proportion	0.31	0.48	0.21
d_N/d_S branches (blue)	0	0.1	ω_3=0.65
d_N/d_S branches (red)	0	0.1	ω_4=0.9
Model D vs M3 (ω_3=ω_4): P=0.41 (χ^2, d.f.=1)			
Model D vs D neutral (ω_3=1): P=0.75 (χ^2, d.f.=1)			

Supplementary Table 2: PAML statistical analysis showing no significant difference in the model comparison between TERF2 TRFH domain sites. Complete TERF2 TRFH domain sequences were retrieved for the following 18 vertebrate species and used for this analysis: dog, cow, pig, dolphin, megabat, mouse, rat, hamster, guinea pig, rabbit, human, armadillo, opossum, tammar wallaby, chicken, zebra finch, clawed frog and zebrafish.

**Model D results TERF2 dim**			
Site class	0	1	2
Proportion	0.63	0.18	0.19
d_N/d_S branches (blue)	0.01	0.003	ω_3=0.7
d_N/d_S branches (red)	0.01	0.003	ω_4=0.32
Model D vs M3 (ω_3=ω_4): P=0.07 (χ^2, d.f.=1)			
Model D vs D neutral (ω_3=1): P=0.05 (χ^2, d.f.=1)			

Supplementary Table 3: Oligonucleotides used in this study

Sequence motif	primer sequence (5’-->3’)
TTAGGG for	TTAGGGTTAGGGTTAGGGTTAGGGTTAGGGTTAGGGTTAGGGTTAGGGTTAGGGTTAGGG
TTAGGG rev	AACCCTAACCCTAACCCTAACCCTAACCCTAACCCTAACCCTAACCCTAACCCTAACCCT
GTGAGT for	GTGAGTGTGAGTGTGAGTGTGAGTGTGAGTGTGAGTGTGAGTGTGAGTGTGAGTGTGAGT
GTGAGT rev	ACACTCACACTCACACTCACACTCACACTCACACTCACACTCACACTCACACTCACACTC

Supplementary References

1. Dejardin, J. & Kingston, R. E. Purification of proteins associated with specific genomic Loci. *Cell* **136,** 175–186 (2009).
2. Giannone, R. J. *et al.* The protein network surrounding the human telomere repeat binding factors TRF1, TRF2, and POT1. *PLoS One* **5,** e12407 (2010).
3. Grolimund, L. *et al.* A quantitative telomeric chromatin isolation protocol identifies different telomeric states. *Nat Commun* **4,** 2848 (2013).
4. Nittis, T. *et al.* Revealing novel telomere proteins using in vivo cross-linking, tandem affinity purification, and label-free quantitative LC-FTICR-MS. *Mol Cell Proteomics* **9,** 1144–1156 (2010).
5. Lee, O.-H. *et al.* Genome-wide YFP fluorescence complementation screen identifies new regulators for telomere signaling in human cells. *Mol Cell Proteomics* **10,** M110.001628 (2011).
6. Luo, Z. *et al.* TeloPIN: a database of telomeric proteins interaction network in mammalian cells. *Database (Oxford)* **2015** (2015).
7. Yang, Z. PAML 4. phylogenetic analysis by maximum likelihood. *Mol Biol Evol* **24,** 1586–1591 (2007).
